# Antioxidant and Antimicrobial Properties of the Essential Oil and Extracts of *Zanthoxylum alatum* Grown in North-Western Himalaya

**DOI:** 10.1155/2013/790580

**Published:** 2013-05-28

**Authors:** Sanjay Guleria, A. K. Tiku, Apurva Koul, Sahil Gupta, Gurjinder Singh, V. K. Razdan

**Affiliations:** ^1^Natural Product Laboratory, Division of Biochemistry and Plant Physiology, Sher-e-Kashmir University of Agricultural Sciences and Technology, Chatha, Jammu 180009, India; ^2^School of Life Sciences, Jawaharlal Nehru University, New Mehrauli Road, New Delhi 110067, India; ^3^School of Biotechnology, University of Jammu, Jammu 180006, India; ^4^Division of Cancer Pharmacology, Indian Institute of Integrative Medicine (CSIR, India), Canal Road, Jammu 180001, India; ^5^Division of Plant Pathology, Sher-e-Kashmir University of Agricultural Sciences and Technology, Chatha, Jammu 180009, India

## Abstract

The essential oil obtained from the fresh leaves of *Zanthoxylum alatum* was analysed by gas chromatography/mass spectrometry (GC/MS). Fourteen components were identified, and linalool (30.58%), 2-decanone (20.85%), **β**-fenchol (9.43%), 2-tridecanone (8.86%), **β**-phellandrene (5.99%), Sabinene (4.82%), and **α**-pinene (4.11%) were the main components. The EO and methanolic extract of *Z. alatum* exhibited potent antifungal activity against *Alternaria alternata*, *Alternaria brassicae*, and *Curvularia lunata*. The EO also showed significant antibacterial activity against *Bacillus subtilis*, *Micrococcus luteus*, *Staphylococcus aureus*, and *Escherichia coli*. Further, antimicrobial constituents of the EO were isolated by bioautography and preparative thin layer chromatography (PTLC) and identified as **β**-fenchol and linalool using GC/MS analysis. In addition to this, the free radical scavenging activity and antioxidant potential of EO and methanolic extract/fractions of *Z. alatum* were also investigated using *in vitro* assays including scavenging ability against DPPH^•^, reducing power and chelating ability on Fe^2+^ ions. Our results demonstrate that *Z. alatum* could be used as a resource of antioxidant and antimicrobial compounds which may find applications in food and pesticide industries.

## 1. Introduction

Plant diseases caused by microorganisms are responsible for heavy loss of agricultural crops every year. Synthetic chemicals used to control the diseases of crop plants are generally toxic and have negative impact on the environment and human health [1]. Furthermore, there is a heavy risk of development of resistance to these chemicals by disease causing microorganisms [2, 3]. Therefore, there is a need for development of new natural product based antimicrobial compounds which are less toxic and eco-friendly [4, 5]. 

Oxidative stress induced by oxygen radicals is reported to be causative agent of various degenerative diseases like arthritis, cancer arthrosclerosis, diabetes, and Parkinson's disease [6, 7]. Oxidation also causes rancidity in food products, leading to degradation of lipids and proteins, deterioration of flavour, taste, colour, and nutritional quality of the processed food [8]. Although, synthetic antioxidants like BHT and BHA are available in the market, but their use is limited due to the side effects caused by them [9]. It has been shown that natural products present in medicinal plants are inhibitory to the deleterious effects of oxidative stress [10]. Plant essential oils (EOs) and their extracts have extensive use in folk medicines, food flavouring, fragrance, and pharmaceutical industry as they are endowed with antimicrobial, antioxidant, and anti-inflammatory properties [11, 12].

The genus *Zanthoxylum* belonging to family Rutaceae comprises over 200 species, among them *Zanthoxylum alatum* Roxb. which is a medicinal shrub, locally known as “Timber” growing in the valleys of sub-tropical Himalayas [13]. Its fruits branches and thorns are generally used as carminative, stomachic, and remedy for toothache [14]. In India, different parts of the *Z. alatum* are used in Ayurvedic practices for the treatment of skin diseases, abdominal pain, anorexia, and ataxia [15]. In this paper we report the chemical composition, antimicrobial and antioxidant activity of EO and extracts of *Z. alatum* growing in north-western Himalaya. To the best of our knowledge, this study can be assumed as first report on isolation and identification of antimicrobial molecules from *Z. alatum* EO.

## 2. Materials and Methods

### 2.1. Plant Material and Extraction of EO

Fresh leaves from *Z. alatum* were collected and identified at Herbal Garden and Herbarium Research Institute in ISM, Joginder Nagar, District Mandi (HP), India. They were subjected to hydrodistillation for 2 h using a Clevenger-type apparatus for the extraction of EO. The oil was stored at 4°C in the dark until analyzed.

### 2.2. Qualitative and Quantitative Analysis of EO

Analysis of the oil using gas chromatography and mass spectrometry was carried out at Indian Institute of Integrative Medicine (CSIR, India), Canal Road, Jammu, India. A GC-MS 4000 (Varian, USA) system with a HP-5MS agilent column (30 m × 0.25 mm i.d., 0.25 *μ* film thickness). Injector temperature was 280°C. Oven temperature programme used was holding at 50°C for 5 min, heating to 280°C at 3°C/min, and keeping the temperature constant at 280°C for 7 min. Helium was used as a carrier gas at a constant flow of 1.0 mL/min and an injection volume of 0.20 *μ*L was employed. The MS scan parameters included electron impact ionization voltage of 70 eV, a mass range of 40–500 m/z. The identification of components of the essential oil was based on comparison of their mass spectra with those stored in NIST05 library or with mass spectra from literature [16].

### 2.3. Fungal Species and Antifungal Assay

The cultures of agricultural pathogenic fungi, namely, *Alternaria brassicae*, *Alternaria alternata* were obtained from Division of plant Pathology, Sher-e-Kashmir University of Agricultural Sciences and Technology, Chatha, Jammu, India, and *Curvularia lunata* from Type Culture Collection Centre, Indian Agricultural Research Institute (IARI), New Delhi, India. Test component (EO/extract) was added to the sterilized potato dextrose agar (containing 0.5% T20 v/v) in 9 cm petri plates. After preparing the plates containing different concentrations of EO, 5 mm bit of test fungus was inoculated in the centre of the agar plate (mycelial surface of the bit was placed upside down). Plates were incubated in dark at 26°C. The extension diameter (cm) of hyphae from the centre to the side of the dish was measured at 24 h interval, till the growth of fungus in the plate without test component (control) reached the edge of the plate. The experiment was repeated thrice and results were expressed as average of three replicates.

Fungal growth diameter in each plate containing different concentrations of test component was determined to calculate per cent growth inhibition [17].

The antifungal indices were calculated as

(1) $$\begin{array}{c} \text{Antifungal}\;\;\text{index}\;\;\left(\%\right)=\left(1-\frac{{\text{D}}_{\text{a}}}{{\text{D}}_{\text{b}}}\right)\times 100, \end{array}$$

where D_a_ = diameter  of  growth  zone  in  the  experiment dish  (in  cm), D_b_ = diameter  of  growth  zone  in  the  control (in  cm).

### 2.4. Antimicrobial Activity

Bacterial strains, *Bacillus subtilis* MTCC2389, *Staphylococcus aureus* MTCC7443 *Micrococcus luteus* MTCC4821, *Escherichia coli* MTCC2127, and *Pseudomonas aeruginosa* MTCC2642 were obtained from Institute of Microbial Technology (IMTECH, CSIR), Chandigarh, India. Qualitative analysis for screening of antimicrobial activity of EO/methanol extract was carried out by Agar-well diffusion method [18]. 20 mL of sterilized nutrient agar was inoculated with 100 *μ*L of bacterial suspension (10^8^ CFU/mL) and then poured on to sterilized petri plate. The agar plate was left to solidify at room temperature. A well of 6 mm was aseptically bored into the agar plate and 20 *μ*L of the EO (diluted with DMSO, 1 : 1) and methanol extract (2 mg) was added in each well. Chloramphenicol (10 *μ*g) was used as a positive reference to determine the sensitivity of bacteria. The plates were kept at 4°C for 2 h to allow the dispersal and then incubated at 37°C for 24 h.

### 2.5. Determination of MIC by Broth Dilution Technique

Broth dilution technique was used to determine the minimum inhibitory concentration of the EO against five bacterial strains [19]. One millilitre of nutrient broth was kept in each tube and autoclaved. The EO diluted with DMSO (1 : 1) was filtered with (0.22 *μ*m) filter disk before use and then added to each tube to keep the final concentration ranging from 62.5 *μ*g/mL–2000 *μ*g/mL. The test bacterial suspension was added into each tube to yield bacterial density of 10^6^ CFU/mL and the inoculated tubes were incubated at 37°C for 24 h. Tubes containing nutrient broth without EO served as positive control, whereas, without bacteria as negative control. After incubation, 50 *μ*L of 0.2 mg/mL *p*-iodonitrotetrazolium violet (INT) was added in each tube to indicate the bacterial growth. The tubes were again incubated for 30 min at 37°C. Development of pink colour in the tube (due to reduction of dye) indicated the bacterial growth, whereas tubes without colour indicated no active bacterial growth. The lowest concentration at which no bacterial growth was observed (as indicated by colour) corresponded to the minimum inhibitory concentration (MIC). All the assays were performed in triplicate.

### 2.6. Qualitative Antifungal and Antibacterial Activity Assay by Bioautography

Direct bioautography was performed using silica gel 60 F_254_ TLC plates (Merck). Five microlitres of 1 : 10 dilution of EO in dichloromethane were applied to the TLC plates and developed in hexane-ethyl acetate (9 : 1) solvent system.

For assaying antifungal activity, aliquots of 25–50 mL of inoculum spray solution (ca. 3 × 10^5^ conidia/mL) were prepared for test fungi (*A. alternata*) with liquid potato dextrose (potato 200 g, dextrose 20 g, and water to make total volume of litre). 100 mL chromatographic sprayer plate was sprayed lightly (to a damp appearance) three times with spore suspension and incubated for 4 d in a dark moist chamber at 25°C. Fungal growth inhibition appeared as clear zones against a dark background.

For assaying antibacterial activity direct bioautography method was used [20]. TLC plates were developed as mentioned previously. The developed plates were allowed to dry in a stream of air to remove residual solvent which might otherwise inhibit bacterial growth. *Bacillus subtilis* was then sprayed on the TLC plate and incubated at 37°C in humid conditions. After incubation plate was sprayed with 2 mg/mL solution of INT. Clear zones on chromatogram indicated inhibition of growth after incubation.

### 2.7. Isolation of Antimicrobial Constituents

After identification of the inhibition zones on the TLC plate, PTLC was performed by loading the essential oil onto a preactivated silica gel 60 F_254_ coated glass plate (20 × 20 cm, 500 *μ*m thickness) which was developed in *n*-hexane/ethyl acetate (9 : 1, v/v) solvent system. The separated compounds were visualized under UV light (365 and 254 nm) or by spraying with vanillin/sulphuric acid spray reagent. The isolation was carried out by scrapping off the detected zones corresponding to the antimicrobial constituents Za_1_ (*R*_*f*_ = 0.40) and Za_2_ (*R*_*f*_ = 0.61) and transferring them into percolator. The substances were then set free from silica gel by elution with dichloromethane.

### 2.8. Antifungal Activity of Bioactive Molecules Isolated from *Z. alatum*

Different amounts of Za_1_ and Za_2_ were loaded onto the TLC plate and bioautography was performed as described earlier using *A. alternata* as test pathogen. Antifungal activity was determined as MIA of active compounds required for the inhibition of fungal growth on TLC plate.

### 2.9. Identification of Bioactive Compounds

The isolated bioactive compounds corresponding to Za_1_ and Za_2_ were determined using GC/MS analysis using standard compounds, NIST and Wiley libraries, and those reported in the literature [16].

### 2.10. DPPH Radical Scavenging Assay for Essential Oil

Radical scavenging activity was determined using DPPH^•^ method [21]. One mL of different concentrations of the EO or extract was mixed with 1 mL of a 90 *μ*M DPPH^•^ solution in methanol, and final volume was made to 4 mL with methanol. The mixtures were well shaken and kept at 25°C in the dark for 1 h. The absorbance was measured at 517 nm. The radical scavenging activity (RSA) was calculated as a percentage of DPPH^•^ discolouration, using the equation:

(2) $$\begin{array}{c} \%\text{RSA}=\left[\frac{\left(A_{0}-A_{s}\right)}{A_{0}}\right]\times 100, \end{array}$$

where *A*_0_ is the absorbance of the control (containing all reagents except the test compound) and *A*_s_ is the absorbance of test compound. Oil concentration providing 50% inhibition (IC_50_) was calculated from the graph by plotting inhibition % against oil concentration. BHT was used as reference.

### 2.11. DPPH Radical Scavenging Assay for Extracts

In this assay, free radical scavenging activity was determined by measuring the bleaching of purple-coloured methanol solution of DPPH^•^. The radical scavenging activity was determined according to the method of Abe and others [22] with modifications. One millilitre from a 0.5 mM methanol solution of the DPPH radical was mixed to 2.0 mL sample and to this 2.0 mL of 0.1 M sodium acetate buffer (pH 5.5) was added. The mixtures were well shaken and kept at room temperature in the dark for 30 min. The absorbance was measured at 517 nm using a double beam UV-VIS spectrophotometer. Methanol was used as a negative control. BHT was used as standard antioxidant. The radical scavenging activity (RSA) was calculated as a percentage of DPPH^•^ discolouration, using the equation

(3) $$\begin{array}{c} \%\text{RSA}=\left[\frac{\left(A_{0}-A_{s}\right)}{A_{0}}\right]\times 100, \end{array}$$

where *A*_0_ is the absorbance of the control (containing all reagents except the test compound) and *A*_*s*_ is the absorbance of test compound. Extract concentration providing 50% inhibition (IC_50_) was calculated from the graph by plotting inhibition % against extract concentration. BHT was used as reference.

### 2.12. Reducing Power Assay

The reducing power of EO/extract determined the method as described previously [23]. Different concentrations of EO/extract were mixed with 2.5 mL of 0.2 M phosphate buffer (pH 6.6) and 2.5 mL of potassium ferricyanide [K_3_Fe(CN)_6_](1%). The mixture was incubated at 50°C. for 20 min. Aliquots (2.5 mL) of 10% trichloroacetic acid were added to the mixture. The previously mixture was then centrifuged for 10 min at 1036 g. The upper layer of the solution (2.5 mL) was mixed with 2.5 mL of distilled water and 2.5 mL of 1% ferric chloride solution. The absorbance was measured at 700 nm in a double beam UV-VIS spectrophotometer. Increased absorbance of the reaction mixture indicated increased reducing power. The essential oil/fraction concentration providing 0.5 of absorbance (EC_50_) was calculated from the graph of absorbance at 700 nm against EO/extract concentration and compared with those of standard antioxidant.

### 2.13. Chelating Capacity Assay

The chelating effect on ferrous ions of *Z. alatum *EO/extract was estimated by the method of Dinis and others [24] with slight modifications. Briefly, 200 *μ*L of different concentrations of EO/extract and 740 *μ*L of methanol were added to 20 *μ*L of 2 mM FeCl_2_. The reaction was initiated by the addition of 40 *μ*L of 5 mM ferrozine into the mixture, which was then left at room temperature for 10 min before determining the absorbance of the mixture at 562 nm. The ratio of inhibition of ferrozine-Fe^2+^ complex formation was calculated using the equation: % inhibition = [(absorbance of control − absorbance of test sample)/absorbance of control)] × 100.

### 2.14. Estimation of Total Phenolic Content

Total phenolic content was determined according to Folin-Ciocalteu method [25]. Briefly, 0.5 mL of sample was mixed with 0.5 mL of 1 N Folin-Ciocalteu reagent. The mixture was kept for 5 min, followed by the addition of 1 mL of 20% Na_2_CO_3_. After 10 min of incubation at room temperature the absorbance was measured at 730 nm using UV-VIS spectrophotometer. Gallic acid was used as a standard. The concentration of phenolic compounds was calculated according to the following equation that was obtained from the standard gallic acid (5 to 50 *μ*g) graph:

(4) Absorbance=0.0271  gallic  acid  (μg)−  0.253(R2=0.99).

### 2.15. Statistical Analysis

All experiments were carried out in triplicate. Data are expressed as mean ± standard deviation.

## 3. Results and Discussion

### 3.1. Chemical Composition of the EO

Fresh leaves of the plant were subjected to hydrodistillation using a Clevenger-type apparatus and the yellow coloured oil was obtained with yield of 0.08% (w/w). Fourteen compounds comprising 98.4% of the EO were identified by GC/MS analysis. The identified compounds are listed in Table 1 according to their elution order in DB-1 column. The major components of the oil were linalool (30.58%), 2-decanone (20.85%), *β*-fenchol (9.43%), 2-tridecanone (8.86%), *β*-phellandrene (5.99%), Sabinene (4.82%) and *α*-pinene (4.11%). It is evident from previous results that *Z. alatum* oil is rich in linalool and previous researchers have also shown linalool as the major component of EOs from *Zanthoxylum* species [26, 27]. Chemical composition of *Z. alatum* seed EO from northern India has been reported consisting of mainly linalool (71%), limonene (8.2%), *β*-phellandrene (5.7%), and (Z)-methylcinnamate (4.9%) as the major components [28].

### 3.2. Yield of Crude Methanol Extract and Fractions

Percent yield of the crude methanol extract was 20.14%. Crude methanol extract was further partitioned sequentially in different solvents in the order to increas polarity and the yield of different fractions obtained is shown in Table 2. Highest yield was observed in methanol fraction (57.2%) followed by chloroform (31.7%), acetone (6.8%), and ethyl acetate (4.3%) fractions.

### 3.3. *In Vitro* Antibacterial and Antifungal Activities

The results of antibacterial activity assays of the EO and MeOH extract of *Z. alatum* are shown in Table 3. The oil was tested for antibacterial activity against three Gram-negative and two Gram-positive bacteria and was found to be effective against all the tested bacterial strains. The oil strongly inhibited the growth of *M. luteus* (MTCC 4821), *S. aureus* (MTCC 7443), *E. coli* (MTCC 2127), and *B. subtilis* (MTCC 2389) with zones of inhibition ranging from 15–21 mm. Moderate activity was observed against *P. aeruginosa* (MTCC 2642) with the zone of inhibition of 7 mm. All the bacteria were more sensitive to chloramphenicol as compared to the EO except *M. luteus* (MTCC 4821) which was more sensitive to EO. Methanol extract of *Z. alatum* leaf showed no inhibitory effect against both Gram-positive and Gram-negative bacteria under study at tested amount of 2 mg/well. MIC results indicate that out of the five bacterial strains tested *M. luteus* (MTCC 4821) and *S. aureus* (MTCC 7443) were the most sensitive, with the MIC values 62.5 and 125 *μ*g/mL, respectively. *E. coli* (MTCC 2127) and *B. subtilis* (MTCC 2389) were also sensitive to the oil (MIC value of 250 and 500 *μ*g/mL, resp.). *P. aeruginosa* (MTCC 2642) was the least sensitive to the oil (MIC value > 2000 *μ*g/mL). Antifungal and antibacterial activities have been reported in *Z. armatum* fruit essential oil [29].

**Figure 1:**
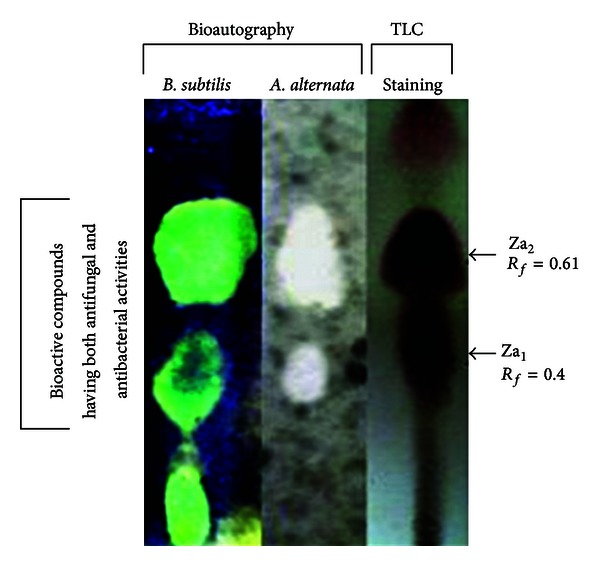
Visualization of antifungal and antibacterial compounds in *Zanthoxylum alatum* leaf essential oil using bioautography.

To evaluate the antifungal activity of EO and methanol extract from *Z. alatum* leaves three agriculturally important phytopathogenic fungi, *A. brassicae, A. alternate*, and *C. lunata,* were selected.  Figure 1 shows the antifungal activities of the EO and methanol extract against test fungal pathogens. On the basis of the results of the antifungal test, the antifungal indices of *Z. alatum* EO were 14.5%, 35.6%, and 42.0% against *A. brassicae*, *A. alternate*, and *C. lunata*, respectively, and IC_50_ values were 2868, 1623, and 1322 *μ*g/mL, respectively (Table 4). Similarly antifungal indices of methanol extract were 47.4% and 51.4% against *A. alternata* and *C. lunata*, respectively, and IC_50_ values were 1071 and 948 *μ*g/mL, respectively (Table 4). It is clear that in comparison with the IC_50_ values, both EO and methanol extract showed higher antifungal activity against *C. lunata* followed by *A. alternata.* Further antifungal indices showed higher efficacy of methanol extract than EO against all the test fungal pathogens. Antifungal activity of crude methanol extract could be attributed to the presence of phenolic and flavonoid compounds [30, 31]. No antifungal activity was observed in the methanol extract at the highest test concentration of 2 mg/mL against *Alternaria brassicae*. EOs are complex mixtures obtained by distillation of a large number of volatile compounds from the plant. EOs rich in oxygenated monoterpenes have been shown to possess antifungal and antibacterial activities [32, 33]. Many researchers have investigated the antifungal and antibacterial activities of individual chemical constituents of the EOs such as *β*-caryophyllene, caryophyllene oxide, and linalool [34, 35]. The compounds which are present in small amount such as *β*-pinene, sabina ketone, and *α*-selinene in *Z. alatum* EO may also contribute to antimicrobial activity either directly or in synergy with some other bioactive compounds. Here we considered it pertinent to identify for the first time the active compounds present in *Z. alatum* EO and investigate their fungitoxic and antibacterial activity.

### 3.4. Antimicrobial Compounds from *Z. alatum* EO

Antifungal and antibacterial compounds of the EO were observed on TLC plate using direct bioautography. They were further isolated using repeated preparative TLC and identified by GC/MS analysis. Bioautography procedure enables the evaluation of plant extracts and EOs against human and plant pathogens [36]. For isolation of antibacterial compounds bioautography was performed using *B. subtilis* as test organism. Similarly *A. alternata* and *C. lunata* were used as test organisms for isolation of antifungal compounds from *Z. alatum* essential oil. Direct bioautography for observing antibacterial activity showed three inhibition zones at *R*_*f*_ values 0.15, 0.40, and 0.61. Similarly when bioautography was performed for observing antifungal activity two inhibition zones at *R*_*f*_ values 0.40 and 0.61 were observed marked as Za_1_ and Za_2_ (Figure 1, Table 5). Intriguingly, compounds corresponding to *R*_*f*_ values 0.40 and 0.61 showed both antifungal and antibacterial activities. The EO of *Z. alatum* was further subjected to repeated PTLC for isolation of compounds showing both antifungal and antibacterial activities. PTLC of the essential oil yielded 3.2 mg of Za_1_ and 5.7 mg of Za_2_. The previous pure compounds were further subjected to bioautography for determination of minimum inhibitory amount (MIA) against *A. alternata* and *C. lunata.* The results of previous investigation showed that Za_1_ possessed stronger fungitoxic activity against *A. alternata* (MIA = 25.3 *μ*g) and *C. lunata* (MIA = 10.0 *μ*g) than Za_2_ with MIA value of 29.4 *μ*g and 41.5 *μ*g against *A. alternata* and *C. lunata*, respectively (Table 6). Further both the antifungal compounds have different response against both the test organisms. Za_1_ was more effective against *C. lunata*, whereas Za_2_ was active against *A. alternata*.

**Figure 2:**
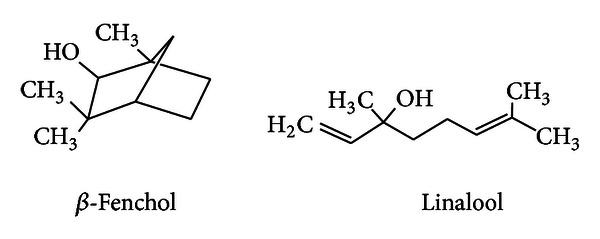
Chemical structures of compounds having antifungal and antibacterial activities.

The bioactive constituents of the *Z. alatum* EO corresponding to Za_1_ and Za_2_ were identified as *β*-fenchol and linalool using GC-MS analysis (Figure 2). In our previous work, we have reported antifungal activity of *β*-fenchol isolated from *Eucalyptus tereticornis* EO against *A. alternate* [32].

### 3.5. Antioxidant Activity of the EO and Extracts of *Z. alatum*

The results of the antioxidant activity determined by three test assays, namely, DPPH radical scavenging, reducing power, and metal ion chelating activity are shown in Table 7. In the present study, the capacity of the EO and extract samples to scavenge DPPH radical and their reducing power was determined on the basis of their concentration providing 50% inhibition (IC_50_). Further, their capacity to chelate Fe^2+^ metal ion was determined at 0.5 mg/mL concentration. BHT, BHA, and quercetin were used as controls. Methanol fraction of the plant extract showed the highest radical scavenging activity with an IC_50_ value of 0.044 mg/mL followed by crude methanol extract (IC_50_ = 0.067 mg/mL) and acetone fraction (IC_50_ = 0.086 mg/mL). The EO and nonpolar fractions, namely, chloroform and ethyl acetate showed relatively low antioxidant activity in this assay. The radical scavenging activity of the leaf extract and fractions of *Z. alatum* may be due to its phenolic compounds as their hydroxyl groups confer radical scavenging ability [37].

Reducing power of plant extracts or EOs may serve as indicator of their antioxidant potential [38]. In this test, there is a change in the colour of test solution from yellow to green depending on the reducing power of test sample. The presence of reductants in the solution causes the reduction of the Fe^3+^/ferricyanide complex to ferrous form. The reducing power of crude extract, its derived fractions, EO, and reference compounds BHT and BHA was dose dependent and increased with increasing concentration. As shown in Table 7, the reducing power is in the order: methanol fraction (IC_50_ = 0.3 mg/mL) > crude extract (IC_50_ = 0.39 mg/mL) > acetone fraction (IC_50_ = 0.96 mg/mL) > ethyl acetate fraction (IC_50_ = 1.15 mg/mL) > chloroform fraction (IC_50_ =1.47 mg/mL) > essential oil (IC_50_ = 11.9 mg/mL). It is interesting and worthy mentioning here that the methanol fraction which had the highest reducing power also possessed the highest radical scavenging activity. 

Crude extract, its derived fractions and EO of *Z. alatum* leaves showed metal ion chelating activity. As shown in Table 7 ethyl acetate fraction showed a better metal ion chelating effect with 60.1% chelating capacity at 0.5 mg/mL, followed by crude extract (43.8%), methanol fraction (42.2%), EO (42.1%), chloroform fraction (41.2%), and acetone fraction (40.5%). The results indicate that *Z. alatum* leaf extracts and essential oil showed good chelating activity.

There are very few reports in the literature about antioxidant activity of linalool as the major component of the EO. Recently Ebrahimabadi and others [39] have shown poor antioxidant activity in linalool in *β*-carotene/linoleic acid test, whereas no radical scavenging activity was reported in DPPH assay. Thus antioxidant activities observed for the* Z. alatum* EO could be attributed to the remaining chemical constituents. 

### 3.6. Total Phenolic Content of *Z. alatum* Leaf Extract and EO

Total phenolics in EO, crude extract and its derived fractions were determined according to Folin-Ciocalteu method and expressed as GAE. As shown in Table 2. The highest amount of total phenolics was observed in methanolic fraction (383.5 mg of GAE/g) followed by crude extract (366.3 mg of GAE/g), acetone fraction (187.6 mg of GAE/g), ethyl acetate fraction (155 mg of GAE/g), and chloroform fraction (139.5 mg of GAE/g). Least phenols were observed in EO (19.3 mg of GAE/g). Several workers have reported that phenolic content of plants is related to their antioxidant activity [40–42]. Phenolic compounds act as reducing agents, hydrogen donors, and singlet oxygen quenchers due to their redox properties [25]. The aforesaid results demonstrate that the free radical scavenging effect (1/IC_50_) of essential oil, crude extract and its derived fractions of *Z. alatum* correlates closely with their phenolic contents (*r* ^2^ = 0.86).

## 4. Conclusions

In conclusion, we investigated the antifungal activities of EO and methanol extract from leaf tissues of *Z. alatum* growing in north-western Himalaya against three phytopathogenic fungi, which had not been reported previously. EO from *Z. alatum* also showed significant antimicrobial activity against Gram-negative bacteria like *M. luteus* and *E. coli *and Gram-positive bacteria, *S. aureus*, and *B. subtilis*. Further, antimicrobial compounds were isolated and identified from the EO as *β*-fenchol and linalool. The results of antioxidant activity tests show that methanol extract and derived fractions of *Z. alatum* should be treated as efficient radical scavenger and reductant as compared to EO. 

## Figures and Tables

**Table 1:** Percent composition of leaf essential oil from *Zanthoxylum alatum*.

S. No.	Compound	RT [min]	Percent (%)	Mode of identification
1	*α*-Pinene	10.170	4.11	GC-MS
2	Sabinene	12.063	4.82	GC-MS
3	*β*-Pinene	12.173	1.31	GC-MS
4	t-Butylbenzene	14.566	2.31	GC-MS
5	*β*-Phellandrene	14.764	5.99	GC-MS
6	Linalool	18.408	30.58	GC-MS
7	4-Terpineol	22.094	2.36	GC-MS
8	Sabina ketone	22.550	0.94	GC-MS
9	*β*-Fenchol	22.746	9.43	GC-MS
10	2-Decanone	27.571	20.85	GC-MS
11	Caryophyllene	32.956	2.76	GC-MS
12	2-Tridecanone	36.119	8.86	GC-MS
13	*α*-Selinene	38.773	0.53	GC-MS
14	Caryophyllene oxide	39.518	2.23	GC-MS

RT: retention time.

**Table 2:** Relative proportion of solvent fractions obtained during fractionation of crude methanolic leaf extract of *Zanthoxylum alatum* expressed as percent of total amount of crude extract used for the fractionation.

Solvent fractions	Percent yield
Chloroform	31.7 ± 0.98
Ethyl acetate	4.3 ± 0.05
Acetone	6.8 ± 0.09
Methanol	57.2 ± 1.78

Values are given as mean ± SD (*n* = 3).

**Table 3:** Antibacterial activity of the essential oil and methanol extract of *Zanthoxylum alatum*.

Microorganism	Zone of inhibition (mm)			MIC (*μ*g/mL)
	Essential oil^a^	MeOH extract^b^	CF	Essential oil
*B. subtilis* MTCC2389	15	—	19	500
*M. luteus* MTCC4821	21	—	20	62.5
*S. aureus* MTCC7443	19	—	21	125.5
*E. coli* MTCC2127	18	—	24	250
*P. aeruginosa* MTCC2642	7	—	18	>1000

Diameter of inhibition zone includes the diameter of the well (6 mm); ^a^essential oil (2 *μ*L well^−1^); ^b^MeOH extract (2 mg well^−1^); standard antibiotic: CF: chloramphenicol (10 *μ*L well^−1^); MIC: minimum inhibitory concentration. —: not detected.

**Table 4:** Percent antifungal index and IC_50_ values of essential oil and methanolic extracts from leaves of *Zanthoxylum alatum* against three crop infecting fungi.

Pathogens	% Antifungal index		Antifungal activity [IC_50_ (*μ*g/mL)]	
	Essential oil	MeOH extract	Essential oil	MeOH extract
*Alternaria alternata *	35.6 ± 1.49	47.4 ± 1.18	1623 ± 41.5	1071 ± 26.2
*Alternaria brassicae *	14.5 ± 0.36	—	2868 ± 61.2	—
*Curvularia lunata *	42.0 ± 1.63	51.2 ± 1.47	1322 ± 24.9	948 ± 21.8

Values are given as mean ± SD (*n* = 3). —: not active at test concentration.

**Table 5:** Antifungal activity of essential oil isolated from *Zanthoxylum alatum* leaves against *Alternaria alternata*, *Curvularia lunata,* and *B. subtilis* using bioautography.

Component	*R*_*f*_ value	Diameter of inhibition zone (mm)		
		*Alternaria alternata *	*Curvularia lunata *	*B. subtilis *
Za_1_	0.40	13.0 ± 0.42	11.0 ± 0.30	20.0 ± 0.69
Za_2_	0.61	18.0 ± 0.38	22.0 ± 0.72	26.5 ± 0.48

Values are given as mean ± SD (*n* = 3).

**Table 6:** Antifungal activity of compounds isolated from *Zanthoxylum alatum* essential oil against *Alternaria alternata* and *Curvularia lunata*.

Component	Minimum inhibitory amount [*μ*g]^a^	
	*Alternaria alternata *	*Curvularia lunata *
Za_1_, *β*-fenchol	25.3 ± 0.28	10.0 ± 0.09
Za_2_, Linalool	29.4 ± 0.37	41.5 ± 0.31

Values are given as mean ± SD (*n* = 3). ^a^Minimum inhibitory amount (MIA) of active constituents required for the inhibition of fungal growth on TLC plate in bioautography assay.

**Table 7:** Antioxidant activity and total phenolic content of the essential oil and methanol extract of *Zanthoxylum alatum*.

Sample	DPPH radical scavenging IC_50_ (mg/mL)	Reducing power IC_50_ (mg/mL)	% Chelation power at 0.5 mg/mL	TPC (mg g^−1^ GAE)
Essential oil	6.04 ± 0.08	11.9 ± 0.29	42.1 ± 1.03	19.3 ± 0.34
Crude methanolic extract	0.067 ± 0.002	0.39 ± 0.01	43.8 ± 1.92	366.3 ± 15.3
Chloroform fraction	0.155 ± 0.004	1.47 ± 0.04	41.2 ± 0.47	139.5 ± 4.03
Ethyl acetate fraction	0.51 ± 0.01	1.15 ± 0.03	60.1 ± 2.71	155.8 ± 7.62
Acetone fraction	0.086 ± 0.003	0.96 ± 0.02	40.5 ± 0.98	187.6 ± 6.09
Methanolic fraction	0.044 ± 0.001	0.3 ± 0.001	41.2 ± 1.02	383.5 ± 11.4
BHT	0.013 ± 0.000	0.14 ± 0.001	—	—
BHA	0.01 ± 0.000	0.11 ± 0.003	—	—
Quercetin	—	—	42.7 ± 0.80	—

Values are given as mean ± SD (*n* = 3).
